# Anthropogenic Noise Aggravates the Toxicity of Cadmium on Some Physiological Characteristics of the Blood Clam *Tegillarca granosa*

**DOI:** 10.3389/fphys.2019.00377

**Published:** 2019-04-03

**Authors:** Wei Shi, Yu Han, Xiaofan Guan, Jiahuan Rong, Xueying Du, Shanjie Zha, Yu Tang, Guangxu Liu

**Affiliations:** College of Animal Science, Zhejiang University, Hangzhou, China

**Keywords:** anthropogenic noise, cadmium, *Tegillarca granosa*, physiological characteristics, neurotoxic

## Abstract

Widespread applications of cadmium (Cd) in various products have caused Cd contamination in marine ecosystems. Meanwhile, human activities in the ocean have also generated an increasing amount of noise in recent decades. Although anthropogenic noise and Cd contaminants could be present simultaneously in marine environments, the physiological responses of marine bivalve mollusks upon coexposure to anthropogenic noise and toxic metal contaminants, including Cd remain unclear. Therefore, the combined effects of anthropogenic noise and Cd on the physiological characteristics of the blood clam *Tegillarca granosa* were investigated in this study. The results showed that 10 days of coexposure to anthropogenic noise and Cd can enhance adverse impacts on metabolic processes, as indicated by the clearance rate, respiration rate, ammonium excretion rate, and O:N ratio of *T. granosa*. In addition, both the ATP content, ATP synthase activity and genes encoding important enzymes in ATP synthesis significantly declined after coexposures to anthropogenic noise and Cd, which have resulted from reduced feeding activity and respiration. Furthermore, the expressions of neurotransmitter-related genes (MAO, AChE, and mAChR3) were all significantly down-regulated after coexposure to anthropogenic noise and Cd, which suggests an enhanced neurotoxicity under coexposure. In conclusion, our study demonstrated that anthropogenic noise and Cd would have synergetic effects on the feeding activity, metabolism, and ATP synthesis of *T. granosa*, which may be due to the add-on of stress responses and neurotransmitter disturbances.

## Introduction

As a byproduct of the zinc, lead and copper refinery, cadmium (Cd) has been recognized as one of the most dangerous toxic metals for many years (Järup, 2003; Kim et al., 2014). The widespread applications of Cd in both consumer and industrial products, such as plastics, ceramics, glass and vehicle tires, have resulted in the consistent presence of Cd contamination in marine ecosystems, which was reported to be as high as 50 μg/L in several heavily polluted areas (Vinarao et al., 2014). Due to its intrinsic ionic similarity to calcium, Cd can be accidentally ingested by marine bivalves and enter into their cells through calcium channels (Vercauteren and Blust, 1999; Shi et al., 2018a), which would subsequently provoke a series of physiological responses, such as a decreased filtration rate, a hampered metabolism, and an altered sex ratio (Liu et al., 2014; Peng et al., 2015a; Shi et al., 2016; Wu et al., 2017). More importantly, many researches have proven that the harmful effects of Cd may occur at a much lower concentration than previously estimated (Zhang et al., 2008).

In the last few decades, human activities have not only brought chemical pollution but also generated an increasing amount of anthropogenic noise in both the open ocean and coastal areas (Peng et al., 2015b). Anthropogenic noise emitted from various ways, such as facility construction, resource exploration and maritime transportation, has led to a new type of pollution, noise pollution (Engås et al., 1996; Vasconcelos et al., 2007). Compared with other environmental disturbances, noise pollution is considered to be extremely harmful because of its universal and uncontrollable characteristics (André, 2009). Anthropogenic noise can directly or indirectly affect a wide variety of marine organisms by disturbing their biological processes and physiological functions, including acoustic communication (Codarin et al., 2009), auditory sensitivity (Popper et al., 2005; Codarin et al., 2009), individual behavior (Popper et al., 2003; Bruintjes and Radford, 2013), and population distribution (Lagardère, 1982; Soto et al., 2013). However, to date, only a few acoustic studies have been conducted with marine invertebrates, especially bivalve mollusks (Peng et al., 2015b). Limited studies have shown that exposure to anthropogenic noise could lead to physiological alterations such as hampered metabolism in marine invertebrates (Peng et al., 2016). For example, an altered O:N ratio and the expression of metabolism-related genes were detected in razor clams, *Sinonovacula constricta*, in response to anthropogenic noise at intensities of ∼80 and ∼100 dB re 1 μPa (Peng et al., 2016).

Anthropogenic noise and Cd contaminants could be present simultaneously in marine environments, especially in polluted coastal areas. Inhabiting the coastal zone, many marine bivalve species are often challenged by multiple environmental stressors (Rocha et al., 2015; Shi et al., 2018a,b; Guan et al., 2018). However, to date, little is known about the physiological responses of marine bivalve mollusks upon coexposure to anthropogenic noise and toxic metal contaminants. To the best of our knowledge, only one recent study investigated the synergetic impacts of anthropogenic noise and trace metal contamination in bivalve mollusks (Charifi et al., 2018). The results showed that compared to that of the control without ship noise, coexposure to cargo ship noise (150 dB re 1 μPa) and waterborne Cd (0.5 μg/L) led to 58.97% reduction in bioaccumulation of Cd in gills and resulted in a decrease in the growth rate of the oyster *Magallana gigas* (Charifi et al., 2018).

Although it remains unclear in marine invertebrates, since the neuroendocrine alterations were often reported along with the physiological adverse impacts in marine vertebrates (Romano et al., 2004; Anderson et al., 2011), it is generally accepted that noise may cause physiological impacts by affecting the neuroendocrine regulation pathway. Similarly, the neurotoxicity of Cd has been well studied in a variety of organisms (Gabbiani et al., 1967; Méndez-Armenta and Ríos, 2007). Therefore, theoretically, coexposure to noise and Cd may show an add-on or an offset effect on the physiological responses, such as metabolism, of an organism through their synergetic impacts on neuroendocrine regulation. However, whether this speculation holds true in bivalve mollusks needs to be verified by empirical data.

As an important aquaculture bivalve species, the blood clam, *Tegillarca granosa* is naturally distributed in the Indo-Pacific region (Liu et al., 2014; Shao et al., 2016). Due to its ecological importance in sediment nutrient cycling and ecosystem carbon flow, many studies have been performed on various aspects of *T. granosa* (Liu et al., 2016; Shi et al., 2017a,b; Zhao et al., 2017). However, the synergetic impacts of noise and Cd on the metabolism of bivalve mollusks, including blood clams, remain unknown to date. Therefore, to obtain a better understanding of the physiological responses of bivalve mollusks to coexposure to Cd and simulated anthropogenic noise, the clearance rate, respiration rate, ammonium excretion rate, O:N ratio, ATP content, activities of ATP synthases, activity of AChE, and expression of neurotransmitter- and ATP synthesis-related genes of *T. granosa* were investigated in this study. The data obtained could help the community to further understand the potential risk of emerging pollution in marine environments.

## Materials and Methods

### Collection and Acclimation of Bivalves

Specimens of adult *T. granosa* (mean ± SE, shell length of 18.23 ± 1.34 mm) were collected from Yueqing Bay (28° 280′ N and 121°110′ E), Zhejiang, China in June 2018. To obtain the background concentration of Cd, seawater was sampled and analyzed in triplicate following the methods described by Shi et al. (2018a). The background concentration of Cd was found to be under the detection limits < 0.01 μg/L). After cleaning off the epizoa, clams were acclimatized for 10 days in a 1000 L indoor tank with filtered seawater (temperature 21.4 ± 1.2°C, pH 8.09 ± 0.03, salinity 20.7 ± 0.1‰) before the experiment. During the acclimation process, the clams were fed twice daily with the microalgae *Tetraselmis chuii* at a rate of 5% of the tissue dry weight, and half the volume of the seawater was replaced with fresh filtered seawater daily. No mortality occurred during this experiment.

### Exposure Experiments

CdCl_2_ (>99% purity) was purchased from Aladdin Chemical Co, China. Stock solutions were prepared in deionized water at 1 M, a concentration high enough to prevent weighing errors and salinity fluctuation during the adding of stock solution to obtain the desired exposure concentrations (Shi et al., 2016). On the basis of previous studies (Chan, 1995), 50 μg/L of Cd^2+^ was chosen in this study to simulate the Cd concentration in heavily polluted coastal areas. The sound broadcast system was composed of an underwater loudspeaker (UW-30, Electro-Voice^®^, Indiana, United States; frequency response 0.1–10 kHz; power-handling capacity 30 watts) connected to a power amplifier player (AV-296, SAST^®^, Guangdong, China; power-handling capacity 150 watts) as described in our previous study (Peng et al., 2016). According to preliminary survey results and reported data (Arveson and Vendittis, 2000; Zou et al., 2004), underwater sound levels of ∼70 and ∼100 dB re 1 μPa were used in this study to simulate sound levels with different degrees of anthropogenic noise input. An ambient aeration sound level of the culture system without any addition of anthropogenic sound input was used as a control. A downloaded pile-driving noise record reported in a previous study was used in this study as the source of anthropogenic sound input (Solan et al., 2016).

In the present study, three experimental groups and one control group were set up as follows: (1) control group without Cd and anthropogenic sound input, (2) Cd treatment group with 50 μg/L Cd^2+^ without anthropogenic sound input, (3) coexposure group with 50 μg/L Cd and 70 dB re 1 μPa anthropogenic noise input, and (4) coexposure group with 50 μg/L Cd and 100 dB re 1 μPa anthropogenic noise input. After acclimation, 480 clams were randomly assigned to 12 individual 160 L buckets (4 treatments × 3 replicate tanks) containing 50 cm deep filtered seawater with slight aeration. The submersible loudspeaker was suspended in the center position of the bucket at a depth of 20 cm below the water surface and oriented to the floor of the bucket to generate the experimental noise effect. The concentrations of Cd^2+^ in each treatment were measured using a graphite furnace atomic absorption spectrophotometer every 2 days during the experiment, as described previously (Shi et al., 2018a; Table 1). The action acoustic conditions during the experiment were measured using acoustics recording units with a bioacoustics recorder (Song Meter SM2+, Wildlife Acoustics^®^, MA, United States; 96 kHz sampling rate). The working sound pressure levels were measured with a hydrophone near the bottom of the tank at 5, 15, and 25 cm away from the center (Table 2). Then, acoustic analyses were performed using Soundscape Analysis Software SACS V1.0 (Register number: 2014SR216788) in MATLAB R2013a (The Math-Works Inc., United States) following the method described previously (Peng et al., 2016). The experiment lasted for 10 days, and no individual mortality was observed throughout the experimental period. The clams were fed with *T. chuii* as mentioned above, and the whole volume of seawater was replaced daily with newly added Cd^2+^ at the designed concentration after feeding.

**Table 1:** The working sound pressure levels (dB re 1 μPa) in the experimental setup at different measuring positions.

Distance to the center	5 cm	15 cm	25 cm
Control	54.53^a^ (52.77∼57.23)	54.09^a^ (52.38∼56.73)	53.45^a^ (51.90∼55.97)
Cd treatment	53.91^a^ (52.99∼56.74)	53.57^a^ (52.47∼56.03)	52.95^a^ (51.75∼55.77)
Co-exposure with 70 dB re 1 μPa noise input	73.78^a^ (70.85∼75.09)	72.15^a^ (69.62∼74.38)	71.52^a^ (68.79∼73.83)
Co-exposure with 100 dB re 1 μPa noise input	101.56^a^ (93.55∼108.05)	100.86^a^ (92.89∼107.57)	98.36^a^ (89.94∼104.37)

*Significant differences at different positions of each experimental setup (data in the same line) are indicated by different superscripts.*

**Table 2:** Waterborne Cd^2+^ concentrations measured for the different groups.

Group	Control	Cd treatment	Co-exposure with 70 dB re 1 μPa noise input	Co-exposure with 100 dB re 1 μPa noise input
Concentration (μg/L)	Not detected	49.72 ± 3.5^a^	50.21 ± 4.3^a^	49.37 ± 2.66^a^

*Significant differences are indicated by different superscripts.*

### Physiological Measurements

#### Clearance Rate

After 10 days of exposure, the clams were fasted for 12 h to empty their digestive tracts prior to the clearance rate measurement. Six blood clam individuals from each replicate tank were randomly selected and transported to a 2 L chamber filled with filtered seawater. Three identical chambers without blood clams were used as the blank control. After approximately 30 min of acclimation, when the valves of the individuals were reopened, the microalgae *T. chuii* was added to the chamber to achieve an initial concentration of 2 × 10^5^ cells mL^−1^ (Zhao et al., 2017). The experiment lasted for 2 h. The microalgae cell concentrations at the beginning and end of the measurements were counted three times using a Neubauer hemocytometer (XB-K-25, Anxin Optical Instrument) under a microscope (BX53, Olympus, Tokyo, Japan). The microalgae cell concentrations in the control tanks did not show any significant variation during the measurements. After the measurements, the soft tissues of the clams were dissected and dried in an oven at 70°C for 72 h. The clearance rate was calculated according to previous studies (Zhao et al., 2017):

$$\mathrm{CR}=V\times ({\mathrm{LnC}}_{0}-{\mathrm{LnC}}_{t})/(W\times T)$$

where CR represents the clearance rate (L g^−1^ h^−1^); V represents the filtered seawater volume in the chamber (L); C_0_ represents the initial microalgae concentration (cells mL^−^); Ct represents the microalgae concentration at time T (cells mL^−1^); W represents the dry weight of soft tissues (g); and T is the experimental time (h).

#### Respiration Rate, Ammonium Excretion Rate, and Oxygen to Nitrogen Ratio

Six clam individuals were randomly sampled from each bucket after 10 days of exposure. After 12 h of depuration, these clams were transported into a closed glass respirometer (2 L) filled with oxygen-saturated filtered seawater. After incubation for approximately 30 min, when the valves of the individuals were reopened, the measurements started with the respirometers sealed off for 2 h. Three identical respirometers without clams were used as the blank control. The dissolved oxygen concentrations at the beginning and the end within the respirometers were measured by an oxygen meter (Multi 3410 SET4, WTW, Germany). The concentrations of ammonia produced by the clams were measured by the phenol-hypochlorite method (Solórzano, 1969). The dry weight of the soft tissues was obtained as mentioned above. The respiratory rate and ammonium excretion rate of the blood clams were calculated according to the following formula:

R(E)=V×(C1−C2)/(W×T)

where R(E) represents the respiration (or ammonium excretion) rate (mg g^−1^ h^−1^); V represents the volume of seawater in each respirometer (L); C_1_ and C_2_ represent the dissolved oxygen (or ammonia) concentrations (mg L^−1^) at the beginning and end of the measurement, respectively, W represents the dry weight of the soft tissues (g); and T is the experimental time (h). The ratio of oxygen consumption to ammonia excretion expressed as atomic equivalents (O:N) was calculated to assess the utilization of the different biochemical compositions for energy metabolism (Peng et al., 2016).

#### Measurements of ATP Content and the Activities of ATP Synthases

Six clams from each bucket were randomly sampled to determine their ATP contents and the activities of 6-phosphofructokinase (PFK) and pyruvate kinase (PK) in their whole tissues after 10 days of corresponding treatment. A volume of 0.1–0.3 g of tissue from each individual was homogenized, and then ice-cold saline at quadruple the volume of the tissue was added to each sample. The homogenates were immediately centrifuged at 4°C and 2000 r/min for 10 min. The total protein concentrations of these samples were determined with a commercial kit (P0006, Beyotime Institute of Biotechnology, China) using the Bradford method (Hammond and Kruger, 1988). The collected supernatants were used for the determination of the ATP content and the activities of the ATP synthases.

The amount of ATP in the whole tissue was determined using a commercial ATP assay kit (A095 Nanjing Jiancheng Bioengineering Institute, China) according to the manufacturer’s instructions and expressed as μmol per mg protein. The activities of PFK and PK were measured using commercial kits (A001 and A007, Nanjing Jiancheng Bioengineering Institute, China) with a spectrophotometer (UV-2100, Shanghai Jinghua Instruments, China) at an absorption wavelength of 340 nm following the manufacturer’s protocols. All the enzyme activities were calculated as U per mg protein, where U was defined as the enzyme causing the conversion of 1 μmol of substrate min^−1^.

#### Determination of AChE Activity

After 10 days of treatment, five clams from each bucket were used to determine the activity of AChE in their whole tissues. The activity of AChE was measured using commercial kits (A024, Nanjing Jiancheng Bioengineering Institute, China) with a microplate reader (Thermo Multiskan Go, United States) at an absorption wavelength of 412 nm and expressed as U per mg protein, where U was defined as the amount of enzyme decomposing 1/6 μmol of substrate per mg protein per minute at a temperature of 37°C. The total protein contents of tissues were determined as mentioned above.

#### Gene Expression Analysis

The expression levels of the genes encoding the key modulating enzymes or their receptors, including monoamine oxidase (MAO), AChE, and muscarinic acetylcholine receptor M3 (mAChR3), which encode dopamine (DA), acetylcholinesterase (AChE), and ACh receptors, respectively, were investigated in this study (Gainey and Greenberg, 2003; Hermida-Ameijeiras et al., 2004; Guan et al., 2018). Furthermore, the genes encoding the important modulating enzymes in ATP synthesis, including citrate synthase (CS), dihydrolipoamide dehydrogenase (DLD) and 2-oxoglutarate dehydrogenase (SucA), were also examined (Owen et al., 2002; Koubaa et al., 2013). The total RNA was isolated from the gill tissue of 5 individuals from each bucket after 10 days of exposure as described in our previous study (Shi et al., 2017b). The RNA quality and the concentration were verified by gel electrophoresis and a NanoDrop 1000 UV/visible spectrophotometer (Thermo Fisher Scientific, United States), respectively. First strand cDNA was synthesized from high-quality total RNA (>500 ng/μL) using the PrimeScript RT reagent Kit (TaKaRa, RR037A) following the manufacturer’s instructions. The amplifications were performed in a total volume of 10 μL containing of 5 μL of SYBR Green Master Mix (Q111-2, Vazyme, China), 0.2 μL of each primer (10 μM), 0.2 μL of ROX Reference Dye (Q111-2, Vazyme, China), 1 μL of cDNA template, and 3.4 μL of double-distilled water. Real-time quantitative PCR was conducted on the StepOnePlus Real-Time PCR System (Applied Biosystems, United States) in triplicate according to the following procedure: 95°C for 5 min, followed by 40 cycles (95 °C for 10 s, 60°C for 30 s). A melting curve analysis was used to confirm the specificity and reliability of the PCR products. The 18S rRNA gene was utilized as an internal reference, and the 2^−ΔΔCT^ method was applied to analyze the relative expression levels of the genes investigated. The primers used are listed in Table 3, and all the primers were synthesized by TsingKe Biotech (Beijing, China).

**Table 3:** Primer sequences for the genes used in the real-time PCR analysis.

Gene	Forward primer (5′–3′)	Reverse primer (5′–3′)	Accession no.
*MAO*	GGTCCTGAACTGTGAGTGTCCTTC	GGATGTCATCGTTATTGGAGGAGGT	MH156850
*AChE*	CCTCACTAGGAGTTGTATTTGGGTT	CTTGGGAAGATGTGCTTGATGCTA	MH156845
*mAChR3*	GCCCGTGAGTAACTTCCCATAAACA	CCAGACAACATCGTTCTTCGCAAAT	MH156849
*CS*	CCCGATACACTTGTCAGAGAGAATT	TTGCCTTGCCTTGTTCTAAGAGTAC	MK170247
*DLD*	ACGCATGTAACTTCTGCTCCTA	GGTGCCCTGTCGCTAGAGAA	MK170248
*SucA*	CCTGGTCCACAATCATAGCATGTCT	TTGGATTGGTCAACTGCTGAAGC	MK170249
*18S*	CTTTCAAATGTCTGCCCTATCAACT	TCCCGTATTGTTATTTTTCGTCACT	JN974506.1

#### Statistical Analysis

One-way ANOVAs followed by Tukey’s *post hoc* tests were conducted to compare the clearance rate, respiration rate, ammonium excretion rate, O:N ratio, ATP content and the activities of the enzymes among the experimental groups. For all the analyses, Levene’s test and Shapiro-Wilk’s test were used to verify the homogeneity of the variances and normality, respectively. In cases where these assumptions were not satisfied by the raw data, the data were arcsine square root transformed prior to analysis. The gene expression levels were analyzed using the Duncan multiple range test. All the statistical analyses were carried out using the Origin-Pro 8 software package. All of the data are presented as the mean ± SE, and a *p*-value less than 0.05 indicated a statistically significant difference.

## Results

### Metabolic Responses to Cd Exposure and Anthropogenic Noise

**FIGURE 1:**
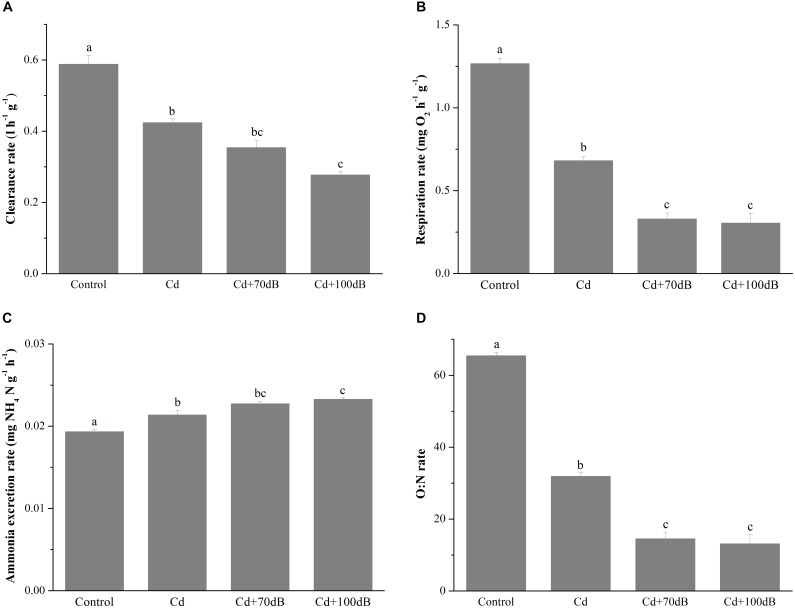
Clearance rates **(A)**, respiration rates **(B)**, ammonium excretion rates **(C)**, and the corresponding oxygen to nitrogen (O:N) ratios **(D)** of the blood clams after 10 days of exposure to a control (without Cd and anthropogenic noise), 50 μg/L Cd, 50 μg/L Cd + 70 dB re 1 μPa anthropogenic noise, and 50 μg/L Cd + 100 dB re 1 μPa anthropogenic noise. Means not sharing the same superscript are significantly different.

Exposure to 50 μg/L Cd alone significantly suppressed the clearance rate of the blood clams (*p* < 0.05) (Figure 1A), which was decreased to approximately 72.2% of that of the control. In addition, coexposure to Cd and anthropogenic noise led to a further decline in the clearance rates (*p* < 0.05), which were reduced to approximately 83.5 and 65.3% of the Cd exposure group for coexposure groups with anthropogenic noise at 70 or 100 dB re 1 μPa, respectively.

Similar results were also observed in the respiration rates of the blood clams after 10 days of exposure to Cd and/or anthropogenic noise (Figure 1B). When exposed to 50 μg/L Cd alone, the respiration rate dropped to approximately 53.8% of that of the control. Coexposure to Cd and anthropogenic noise aggravated the suppression of the respiration rates, which declined to approximately 26.0 and 24.0% of the control for groups coexposed to Cd and 70 or 100 dB re 1 μPa anthropogenic noise, respectively (Figure 1B).

Unlike the clearance rate and respiration rate, the ammonium excretion rates were significantly induced by exposure of the blood clams to Cd and/or anthropogenic noise (*p* < 0.05, Figure 1C). Compared with those of the control, the ammonium excretion rates of the blood clams were significantly elevated to approximately 110% in seawater with 50 μg/L Cd and further increased to 118 and 120% when 70 or 100 dB re 1 μPa anthropogenic noise was copresent with Cd.

Due to both the reduction in respiration rates and the increase in the ammonium excretion rates, the O:N ratios were significantly reduced by Cd exposure alone and coexposure to Cd and anthropogenic noise (Figure 1D, *p* < 0.05). The O:N ratio of clams reduced to approximately 48.7% of the control when exposed to 50 μg/L Cd alone for 10 days and further declined to approximately 22.2 and 20.1% of the control for the groups coexposed to Cd and 70 or 100 dB re 1 μPa anthropogenic noise, respectively (Figure 1D).

### Effects of Cd and Anthropogenic Noise Exposure on ATP Content and the Activities of Synthases

**FIGURE 2:**
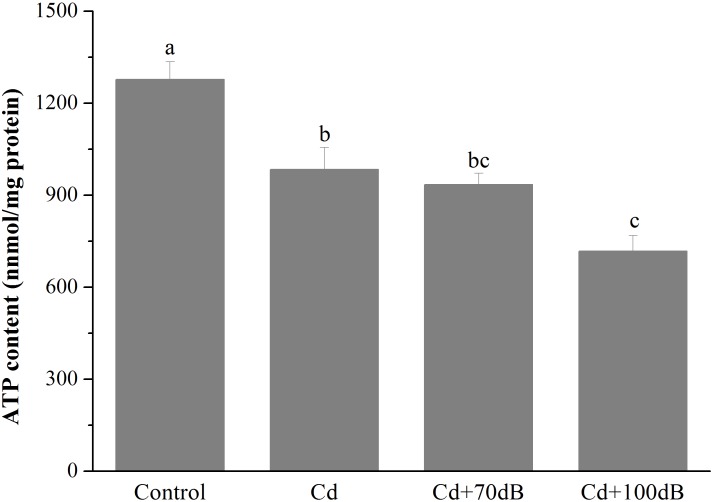
ATP content in the tissues of the blood clams after 10 days of exposure to the control (without Cd and anthropogenic noise), 50 μg/L Cd, 50 μg/L Cd + 70 dB re 1 μPa anthropogenic noise, and 50 μg/L Cd + 100 dB re 1 μPa anthropogenic noise. Means not sharing the same superscript are significantly different.

Both exposure of the clams to Cd alone and coexposure to Cd and anthropogenic noise significantly reduced the ATP contents of the clams (Figure 2, *p* < 0.05). After 10 days of exposure to 50 μg/L Cd alone, the ATP content of the clams was significantly decreased to approximately 77.0% that of the control. Although the ATP content was not further reduced by the copresence of anthropogenic noise at 70 dB re 1 μPa, coexposure to 100 dB re 1 μPa anthropogenic noise significantly aggravated the suppression of ATP content compared to that of the group exposed to Cd alone, which was only approximately 72.9% of that of the group exposed to Cd alone.

**FIGURE 3:**
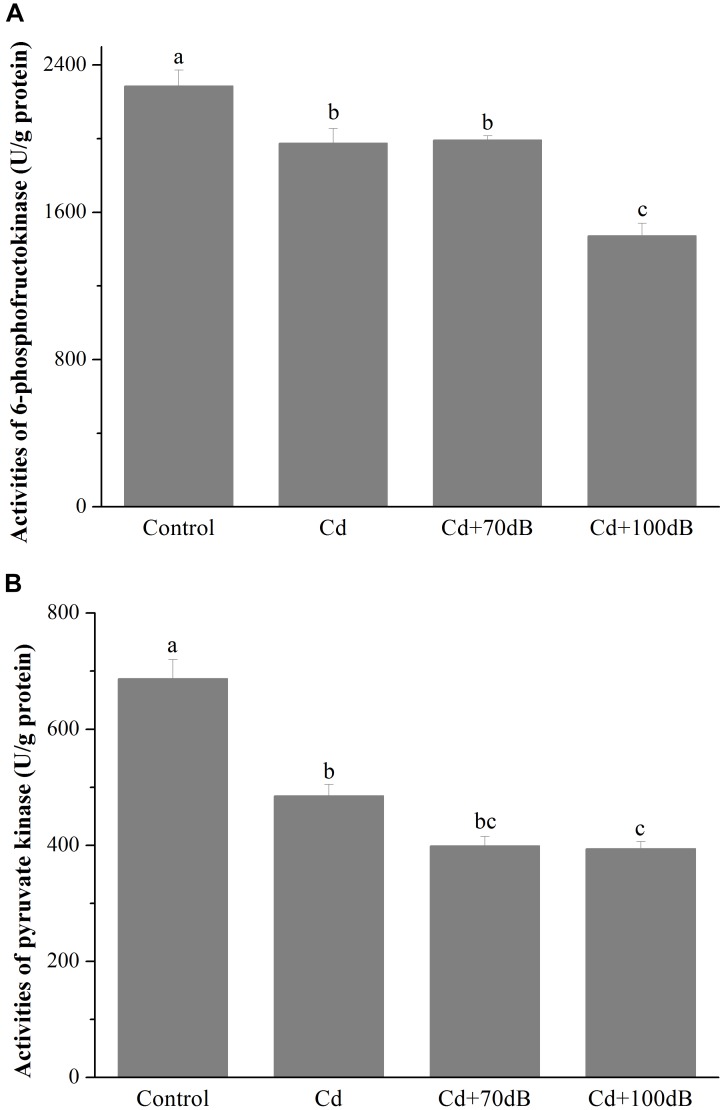
Activities of 6-phosphofructokinase (PFK) **(A)** and pyruvate kinase (PK) **(B)** in the tissues of the blood clams after 10 days of exposure to the control (without Cd and anthropogenic noise), 50 μg/L Cd, 50 μg/L Cd + 70 dB re 1 μPa anthropogenic noise, and 50 μg/L Cd + 100 dB re 1 μPa anthropogenic noise. Means not sharing the same superscript are significantly different.

Similarly, compared to that of the control, the activities of PFK and PK significantly declined to approximately 86.4 and 70.6% of the control, respectively (Figure 3 and *p* < 0.05) when the clams were exposed to 50 μg/L Cd. Although this suppression effect was not significantly aggravated by the copresence of anthropogenic noise at 70 dB re 1 μPa, the activities of PFK and PK were further reduced to 64.4 and 57.3% of that of the control for PFK and PK, respectively, when 100 dB re 1 μPa anthropogenic noise was introduced along with 50 μg/L Cd.

### Effects of Cd and Anthropogenic Noise Exposure on the Activity of AChE

**FIGURE 4:**
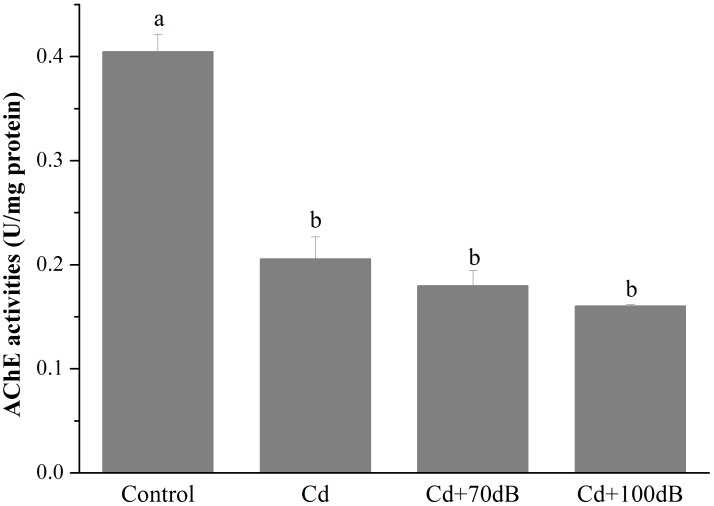
Activities of acetylcholine esterase (AChE) in the tissues of the blood clams after 10 days of exposure to the control (without Cd and anthropogenic noise), 50 μg/L Cd, 50 μg/L Cd + 70 dB re 1 μPa anthropogenic noise, and 50 μg/L Cd + 100 dB re 1 μPa anthropogenic noise. Means not sharing the same superscript are significantly different.

Compared to the control, the activity of AChE in the blood clams was significantly lowered by exposure to Cd and/or anthropogenic noise for 10 days (Figure 4, *p* < 0.05). The blood clams exposed to Cd-contaminated seawater had a significantly lower AChE activity, which was approximately 57.9% of that of the control. However, coexposure to Cd and anthropogenic noise at 70 or 100 dB re 1 μPa did not lead to a further reduction in the activity of AChE when compared to the group exposed to Cd alone.

### Effects of Cd and Anthropogenic Noise Exposure on Gene Expression

**FIGURE 5:**
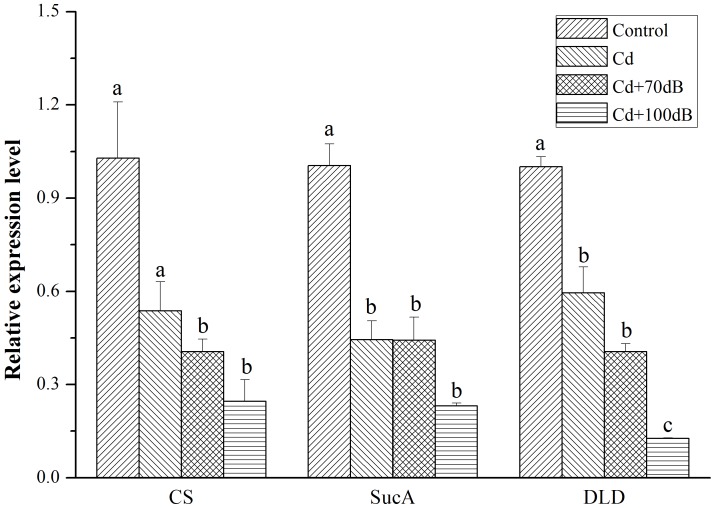
Relative expression levels of the genes in ATP synthesis after 10 days exposure to the control (without Cd and anthropogenic noise), 50 μg/L Cd, 50 μg/L Cd + 70 dB re 1 μPa anthropogenic noise, and 50 μg/L Cd + 100 dB re 1 μPa anthropogenic noise. Means not sharing the same superscript are significantly different.

**FIGURE 6:**
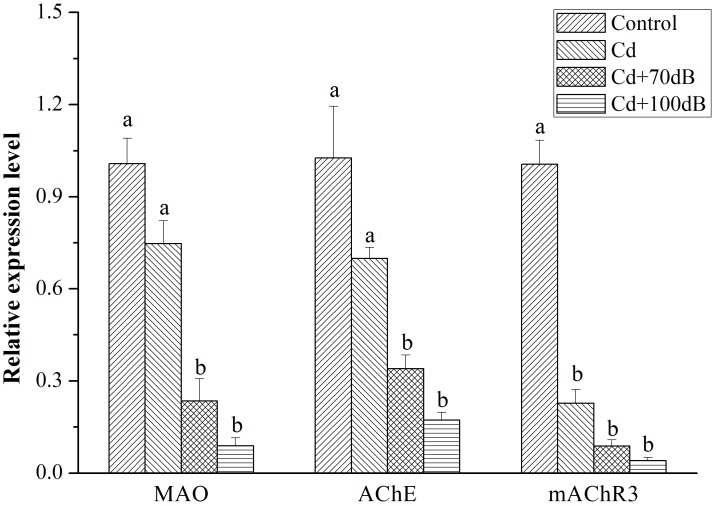
Relative expression levels of the genes encoding neurotransmitter modulatory enzymes and receptors after 10 days of exposure to the control (without Cd and anthropogenic noise), 50 μg/L Cd, 50 μg/L Cd + 70 dB re 1 μPa anthropogenic noise, and 50 μg/L Cd + 100 dB re 1 μPa anthropogenic noise. Means not sharing the same superscript are significantly different.

All three key genes (CS, SucA, and DLD) encoding enzymes for ATP synthesis showed a similar relative expression pattern, with the highest expression detected in the blood clams of the control followed by those in the Cd exposure group and then the lowest expression in the coexposure groups (Figure 5, *p* < 0.05). In addition, when coexposed to Cd and anthropogenic noise, the expression levels of SucA and DLD significantly declined with the increase in sound levels of the anthropogenic noise investigated. Similarly, the downregulation of neurotransmitter-related genes (MAO, AChE and mAChR3) was significantly (*p* < 0.05) aggravated by the copresence of anthropogenic noise (Figure 6, *p* < 0.05).

## Discussion

As a benthic filter feeder, the blood clam, similar to many other sessile marine bivalves, may be challenged by multiple environmental stressors, such as toxic trace metals and anthropogenic noise (Sokolova et al., 2004; Shi et al., 2016; Peng et al., 2016). Constrained by their limited locomotion ability, sessile bivalves have difficulty escaping from these stressors and therefore may be potentially threatened by these stressors. However, the physiological responses of bivalve mollusks to coexposure to anthropogenic noise and toxic trace metals are still largely unknown. In addition, little is known about the molecular mechanism manifesting these physiological impacts.

The results obtained in the present study suggested that coexposure to anthropogenic noise and Cd enhances the adverse impacts on metabolic processes, indicated by the clearance rate, respiration rate, ammonium excretion rate, and O:N ratio of the marine bivalve mollusks. The reduction in the clearance rate detected indicated that the feeding activity of *T. granosa* was significantly suppressed. The Cd-induced reduction in feeding activity was also reported in other marine bivalve species, such as the green-lipped mussel *Perna canaliculus* (Chandurvelan et al., 2012) and the oyster *Crassostrea hongkongensis* (Pan and Wang, 2012). Furthermore, the result that noise exposure reinforces the suppression of the feeding activity of *T. granosa* exposed to Cd in the present study may offer an explanation for the reduction in metal accumulation under the noisy scenario reported, since less Cd will be ingested through feeding (Charifi et al., 2018). In marine bivalves, clearance behavior and respiration are closely linked together, as both processes occur in the water filtration process over the gills during the opening of the valves. It has been shown recently that coexposure to anthropogenic noise and Cd led to an increase in the valve closure duration in the oyster *Magallana gigas* (Charifi et al., 2018). Therefore, both the reduced clearance and respiration rate obtained in this study can result from the suppression of ventilation (Pan and Wang, 2012).

Due to the reduction in respiration and the increase in ammonium excretion, the O:N ratio of the blood clams was significantly downregulated in response to Cd and anthropogenic noise exposures. The O:N ratio is widely considered an indicator of the physiological state of organisms by showing the usage of three substrates (carbohydrates, lipids and proteins) in energy metabolism (Mayzaud and Conover, 1988; Anestis et al., 2010). For example, higher O:N ratios (<30) usually indicate a catabolism of carbohydrates and lipids, while O:N lower ratios (<30) suggest an elevated turnover of proteins under stressful conditions in mussels (Langenbuch and Pörtner, 2002). Therefore, the increase in the ammonium excretion rate along with the reduction in O:N ratio detected suggest that more protein substrates were used to provide energy for the clams, and this phenomenon can be due to the declined energy availability through feeding (Sokolova et al., 2005). In this study, both the ATP content and ATP synthase activity in *T. granosa* significantly declined after stress exposure. In addition, the genes encoding the important enzymes in ATP synthesis were significantly downregulated as well. These ATP synthesis-related results indicated that energy availability was significantly constrained by Cd and anthropogenic noise exposures, which could be a physiological response to the reductions in feeding activity and respiration.

Reduced metabolism is widely adopted as an adaptive mechanism for marine organisms to cope with various environmental stresses, such as toxic metals, ocean acidification and anthropogenic noise (Pan and Wang, 2012; Peng et al., 2016 Zhao et al., 2017). For example, reductions in feeding and respiration may help in reducing the direct contact of the soft tissues to waterborne chemical contaminants such as toxic metals (Pan and Wang, 2012). Although little is known about the physiological impacts of noise on bivalve mollusks, one study has demonstrated that anthropogenic noise may cause razor clams to enter a metabolically inactive state through sending a stress signal via water proton movement sensed by the sensory palps on the mantle and gills (Peng et al., 2016). Therefore, the additive effects of Cd and anthropogenic noise on the metabolism of the blood clams could be simply due to the add-on of their inhibitory impacts.

When exposed to stress conditions, the physiological responses of animals are often regulated through the neuroendocrine pathway (Ravindran et al., 2005; Naqvi et al., 2012). Although the neurotoxic impacts of Cd and anthropogenic noise have been well elaborated in model organisms, the impacts remain unclear in bivalve mollusks especially under combined noise and toxic metal stress. The results obtained in the present study demonstrated that anthropogenic noise can enhance the neurotoxicity of waterborne Cd to bivalves in terms of downregulating the expression of neurotransmitter-related genes. The downregulation of AChE and MAO, which encode the modulatory enzymes for ACh and DA, respectively, could lead to decreased amounts of modulatory enzymes and subsequently constrain the hydrolysis of the corresponding neurotransmitters (Hermida-Ameijeiras et al., 2004; Guan et al., 2018). It has been confirmed that neurotransmitters play important roles in regulating various physiological functions, such as the feeding activity, of many other organisms (Panksepp and Bishop, 1979; Naqvi et al., 2012). For example, a significant inhibitory effect on food intake was observed in Chinese perch, *Siniperca chuatsi*, after 1 h postinjection of 5 μg DA (He et al., 2018). Therefore, exposure to anthropogenic noise may reinforce the physiological impacts of Cd on blood clams through its synergetic effects on disturbing neurotransmitters.

## Conclusion

In conclusion, our study demonstrated that anthropogenic noise and Cd would have synergetic effects on the feeding activity, metabolism, and ATP synthesis of *T. granosa*, which may be due to the add-on of stress responses and neurotransmitter disturbances. This study suggests that although Cd and anthropogenic noise significantly differ in their intrinsic physical and chemical characteristics, the physiological responses provoked are somehow common.

## Author Contributions

WS and GL contributed to conception and design of the experimental plan. WS, YH, XG, JR, XD, YT, and SZ performed the experiments. WS and GL performed the statistical analysis and wrote the manuscript.

## Conflict of Interest Statement

The authors declare that the research was conducted in the absence of any commercial or financial relationships that could be construed as a potential conflict of interest.

## References

[B1] Anderson P. A. Berzins I. K. Fogarty F. Hamlin H. J. Guillette L. J. (2011). Sound, stress, and seahorses: the consequences of a noisy environment to animal health. *Aquaculture* 311 129–138. 10.1016/j.aquaculture.2010.11.013

[B2] André M. (2009). The sperm whale sonar: monitoring and use in mitigation of anthropogenic noise effects in the marine environment. *Nucl. Instrum. Meth. A* 602 262–267. 10.1016/j.nima.2008.12.223

[B3] Anestis A. Pörtner H. O. Karagiannis D. Angelidis P. Staikou A. Michaelidis B. (2010). Response of *Mytilus galloprovincialis* (L.) to increasing seawater temperature and to marteliosis: metabolic and physiological parameters. *Comp. Biochem. Physiol. A* 156 57–66. 10.1016/j.cbpa.2009.12.018 20045485

[B4] Arveson P. T. Vendittis D. J. (2000). Radiated noise characteristics of a modern cargo ship. *J. Acoust. Soc. Am.* 107 118–129. 10.1121/1.428344 10641625

[B5] Bruintjes R. Radford A. N. (2013). Context-dependent impacts of anthropogenic noise on individual andsocial behaviour in a cooperatively breeding fish. *Anim. Behav.* 85 1343–1349. 10.1016/j.anbehav.2013.03.025

[B6] Chan K. M. (1995). Concentrations of copper, zinc, cadmium and lead in rabbitfish (*Siganus oramin*) collected in Victoria Harbour, Hong Kong. *Mar. Pollut. Bull.* 31 277–280. 10.1016/0025-326X(95)00136-B

[B7] Chandurvelan R. Marsden I. D. Gaw S. Glover C. N. (2012). Impairment of green-lipped mussel (*Perna canaliculus*) physiology by waterborne cadmium: relationship to tissue bioaccumulation and effect of exposure duration. *Aquat. Toxicol.* 124-125 114–124. 10.1016/j.aquatox.2012.07.013 22940606

[B8] Charifi M. Miserazzi A. Sow M. Perrigault M. Gonzalez P. Ciret P. (2018). Noise pollution limits metal bioaccumulation and growth rate in a filter feeder, the Pacific oyster *Magallana gigas*. *PLoS One* 13:e0194174. 10.1371/journal.pone.0194174 29617387 PMC5884495

[B9] Codarin A. Wysocki L. E. Ladich F. Picciulin M. (2009). Effects of ambient and boat noise on hearing and communication in three fish species living in a marine protected area (Miramare, Italy). *Mar. Pollut. Bull.* 58 1880–1887. 10.1016/j.marpolbul.2009.07.011 19666180

[B10] Engås A. Løkkeborg S. Ona E. Soldal A. V. (1996). Effects of seismic shooting on local abundance and catch rates of cod ((*Gadus morhua*) and haddock )(*Melanogrammus aeglefinus*). *Can. J. Fish. Aquat. Sci.* 53 2238–2249. 10.1139/f96-177

[B11] Gabbiani G. Baic D. Déziel C. (1967). Toxicity of cadmium for the central nervous system. *Exp. Neurol.* 18 154–160. 10.1016/0014-4886(67)90037-4 6026604

[B12] Gainey L. F. Greenberg M. J. (2003). Nitric oxide mediates seasonal muscle potentiation in clam gills. *J. Exp. Biol.* 206 3507–3520. 10.1242/jeb.00573 12939381

[B13] Guan X. Shi W. Zha S. Rong J. Su W. Liu G. (2018). Neurotoxic impact of acute TiO2 nanoparticle exposure on a benthic marine bivalve mollusk, *Tegillarca granosa*. *Aquat. Toxicol.* 200 241–246. 10.1016/j.aquatox.2018.05.011 29778933

[B14] Hammond J. B. Kruger N. J. (1988). The Bradford method for protein quantitation. *Methods Mol. Biol.* 3 25–32. 10.1385/0-89603-126-8:25 21400151

[B15] He Y. Li L. Liang X. He S. Zhao L. Zhang Y. (2018). Inhibitory neurotransmitter serotonin and excitatory neurotransmitter dopamine both decrease food intake in Chinese perch (*Siniperca chuatsi*). *Fish Physiol. Biochem.* 44 175–183. 10.1007/s10695-017-0422-8 28929258

[B16] Hermida-Ameijeiras Á Méndez-Álvarez E. A. Sánchez-Iglesias S. A. Sanmartıìn-Suárez C. Soto-Otero R. (2004). Autoxidation and MAO-mediated metabolism of dopamine as a potential cause of oxidative stress: role of ferrous and ferric ions. *Neurochem. Int.* 45 103–116. 10.1016/j.neuint.2003.11.018 15082228

[B17] Järup L. (2003). Hazards of heavy metal contamination. *Brit. Med. Bull.* 68 167–182. 10.1093/bmb/ldg032 14757716

[B18] Kim E. Jee J. Steiner H. Cormet-Boyaka E. Boyaka P. (2014). Chronic exposure to cadmium alters gut immune homeostasis and innate immunity (MUC8P.810). *J. Immunol.* 192(1 Suppl.), 198 111.

[B19] Koubaa M. Cocuron J. C. Thomasset B. Alonso A. P. (2013). Highlighting the tricarboxylic acid cycle: liquid and gas chromatography-mass spectrometry analyses of 13 C-labeled organic acids. *Anal. Biochem.* 436 151–159. 10.1016/j.ab.2013.01.027 23399391

[B20] Lagardère J. P. (1982). Effects of noise on growth and reproduction of *Crangon crangon* in rearing tanks. *Mar. Biol.* 71 177–185. 10.1007/BF00394627

[B21] Langenbuch M. Pörtner H. O. (2002). Changes in metabolic rate and N excretion in the marine invertebrate *Sipunculus nudus* under conditions of environmental hypercapnia. *J. Exp. Biol.* 205 1153–1160. 11919274 10.1242/jeb.205.8.1153

[B22] Liu G. Shu M. Chai X. Shao Y. Wu H. Sun C. (2014). Effect of chronic sublethal exposure of major heavy metals on filtration rate, sex ratio, and gonad development of a bivalve species. *B. Environ. Contam. Tox.* 92 71–74. 10.1007/s00128-013-1138-9 24162647

[B23] Liu S. Shi W. Guo C. Zhao X. Han Y. Peng C. (2016). Ocean acidification weakens the immune response of blood clam through hampering the NFκβ and toll-like receptor pathways. *Fish Shellfish Immunol.* 54 322–327. 10.1016/j.fsi.2016.04.030 27109580

[B24] Mayzaud P. Conover R. (1988). O:N atomic ratio as a tool to describe zooplankton metabolism. *Mar. Ecol. Prog. Ser.* 45 289–302. 10.3354/meps045289

[B25] Méndez-Armenta M. Ríos C. (2007). Cadmium neurotoxicity. *Environ. Toxicol. Phar.* 23 350–358. 10.1016/j.etap.2006.11.009 21783780

[B26] Naqvi F. Haider S. Batool Z. Perveen T. Haleem D. J. (2012). Sub-chronic exposure to noise affects locomotor activity and produces anxiogenic and depressive like behavior in rats. *Pharmacol. Rep.* 64 64–69. 10.1016/S1734-1140(12)70731-4 22580521

[B27] Owen O. E. Kalhan S. C. Hanson R. W. (2002). The key role of anaplerosis and cataplerosis for citric acid cycle function. *J. Biol. Chem.* 277 30409–30412. 10.1074/jbc.R200006200 12087111

[B28] Pan K. Wang W. (2012). Reconstructing the biokinetic processes of oysters to counteract the metal challenges: physiological acclimation. *Environ. Sci. Technol.* 6 10765–10771. 10.1021/es302040g 22913643

[B29] Panksepp J. Bishop P. (1979). Neurohumoral and endocrine control of feeding. *Psychoneuroendocrino.* 4 89–106. 10.1016/0306-4530(79)90023-4 42939

[B30] Peng C. Zhao X. Han Y. Shi W. Liu S. Liu G. (2015a). Toxic effects of chronic sub-lethal Cu^2+^, Pb^2+^ and Cd^2+^ on antioxidant enzyme activities in various tissues of the blood cockle, *Anadara granosa*. *J. Res. Sci. Tech.* 12 125–131.

[B31] Peng C. Zhao X. Liu G. (2015b). Noise in the sea and its impacts on marine organisms. *Int. J. Environ. Res. Public Health* 12 12304–12323. 10.3390/ijerph121012304 26437424 PMC4626970

[B32] Peng C. Zhao X. Liu S. Shi W. Han Y. Guo C. (2016). Effects of anthropogenic sound on digging behavior, metabolism, Ca^2+^/Mg^2+^ATPase activity, and metabolism-related gene expression of the bivalve *Sinonovacula constricta*. *Sci. Rep.* 6:24266. 10.1038/srep24266 27063002 PMC4827120

[B33] Popper A. N. Fewtrell J. Smith M. E. Mccauley R. D. (2003). Anthropogenic sound: effects on the behavior and physiology of fishes. *Mar. Technol. Soc. J.* 37 35–40. 10.4031/002533203787537050

[B34] Popper A. N. Smith M. E. Cott P. A. Hanna B. W. Macgillivray A. O. Austin M. E. (2005). Effects of exposure to seismic airgun use on hearing of three fish species. *J. Acoust. Soc. Am.* 117 3958–3971. 10.1121/1.1904386 16018498

[B35] Ravindran R. Rathinasamy S. D. Samson J. Senthilvelan M. (2005). Noise-stress-induced brain neurotransmitter changes and the effect of *Ocimum sanctum* (Linn) treatment in albino rats. *J. Pharmacol. Sci.* 98 354–360. 10.1254/jphs.FP0050127 16113498

[B36] Rocha T. L. Gomes T. Sousa V. S. Mestre N. C. Bebianno M. J. (2015). Ecotoxicological impact of engineered nanomaterials in bivalve molluscs: an overview. *Mar. Environ. Res.* 111 74–88. 10.1016/j.marenvres.2015.06.013 26152602

[B37] Romano T. A. Keogh M. J. Kelly C. Feng P. Berk L. Schlundt C. E. (2004). Anthropogenic sound and marine mammal health: measures of the nervous and immune systems before and after intense sound exposure. *Can. J. Fish. Aquat. Sci.* 61 1124–1134. 10.1139/f04-055

[B38] Shao Y. Chai X. Xiao G. Zhang J. Lin Z. Liu G. (2016). Population genetic structure of the blood clam, *Tegillarca granosa*, along the pacific coast of asia: isolation by distance in the sea. *Malacologia* 59 303–312. 10.4002/040.059.0208

[B39] Shi W. Guan X. Han Y. Guo C. Rong J. Su W. (2018a). Waterborne Cd2+ weakens the immune responses of blood clam through impacting Ca2+ signaling and Ca2+ related apoptosis pathways. *Fish Shellfish Immunol.* 77 208–213. 10.1016/j.fsi.2018.03.055 29609026

[B40] Shi W. Guan X. Han Y. Zha S. Fang J. Xiao G. (2018b). The synergic impacts of TiO2 nanoparticles and 17β-estradiol (E2) on the immune responses, E2 accumulation, and expression of immune-related genes of the blood clam, *Tegillarca granosa*. *Fish Shellfish Immunol.* 81 29–36. 10.1016/j.fsi.2018.07.009 29981881

[B41] Shi W. Han Y. Guo C. Zhao X. Liu S. Su W. (2017a). Immunotoxicity of nanoparticle nTiO2 to a commercial marine bivalve species, *Tegillarca granosa*. *Fish Shellfish Immunol.* 66 300–306. 10.1016/j.fsi.2017.05.036 28522418

[B42] Shi W. Han Y. Guo C. Zhao X. Liu S. Su W. (2017b). Ocean acidification hampers sperm-egg collisions, gamete fusion, and generation of Ca2+ oscillations of a broadcast spawning bivalve, *Tegillarca granosa*. *Mar. Environ. Res.* 130 106–112. 10.1016/j.marenvres.2017.07.016 28750793

[B43] Shi W. Zhao X. Han Y. Che Z. Chai X. Liu G. (2016). Ocean acidification increases cadmium accumulation in marine bivalves: a potential threat to food safety. *Sci. Rep.* 6:20197. 10.1038/srep20197 26795597 PMC4726208

[B44] Sokolova I. M. Evans S. Hughes F. M. (2004). Cadmium-induced apoptosis in oyster hemocytes involves disturbance of cellular energy balance but no mitochondrial permeability transition. *J. Exp. Biol.* 207 3369–3380. 10.1242/jeb.01152 15326213

[B45] Sokolova I. M. Sokolov E. P. Ponnappa K. M. (2005). Cadmium exposure affects mitochondrial bioenergetics and gene expression of key mitochondrial proteins in the eastern oyster *Crassostrea virginica* Gmelin (Bivalvia: Ostreidae). *Aquat. Toxicol.* 73 242–255. 10.1016/j.aquatox.2005.03.016 15935864

[B46] Solan M. Hauton C. Godbold J. A. Wood C. L. Leighton T. G. White P. (2016). Anthropogenic sources of underwater sound can modify how sediment-dwelling invertebrates mediate ecosystem properties. *Sci. Rep.* 6:20540. 10.1038/srep20540 26847483 PMC4742813

[B47] Solórzano L. (1969). Determination of ammonia in natural waters by the phenolhypochlorite method. *Limnol. Oceanogr.* 14 799–801. 10.4319/lo.1969.14.5.0799

[B48] Soto N. A. D. Delorme N. Atkins J. Howard S. Williams J. Johnson M. (2013). Anthropogenic noise causes body malformations and delays development in marine larvae. *Sci. Rep.* 3:2831. 10.1038/srep02831 24088868 PMC3789146

[B49] Vasconcelos R. O. Amorim M. C. Ladich F. (2007). Effects of ship noise on the detectability of communication signals in the Lusitanian toadfish. *J. Exp. Biol.* 210 2104–2112. 10.1242/jeb.004317 17562883

[B50] Vercauteren K. (1999). Uptake of cadmium and zinc by the mussel *Mytilus edulis* and inhibition by calcium channel and metabolic blockers. *Mar. Biol.* 135 615–626.

[B51] Vinarao R. T. Salem G. M. Ragaza R. J. (2014). “Distribution of Cd, Pb, As and Hg in oyster tissue, sediment and water in Lingayen Gulf, Philippines,” in *Molluscan Shellfish Safety*, ed. Sauvé G. (Dordrecht: Springer), 137–154. 10.1007/978-94-007-6588-7_12

[B52] Wu H. Xu L. Yu D. Ji C. (2017). Differential metabolic responses in three life stages of mussels *Mytilus galloprovincialis* exposed to cadmium. *Ecotoxicology* 26 1–7. 10.1007/s10646-016-1741-8 27796687

[B53] Zhang W. Pang F. Huang Y. Yan P. Lin W. (2008). Cadmium exerts toxic effects on ovarian steroid hormone release in rats. *Toxicol. Lett.* 182 18–23. 10.1016/j.toxlet.2008.07.016 18708132

[B54] Zhao X. Shi W. Han Y. Liu S. Guo C. Fu W. (2017). Ocean acidification adversely influences metabolism, extracellular pH and calcification of an economically important marine bivalve, *Tegillarca granosa*. *Mar. Environ. Res.* 125 82–89. 10.1016/j.marenvres.2017.01.007 28188988

[B55] Zou C. Chen D. Hua H. (2004). Study on characteristics of ship underwater radiation noise. *J. Ship Mech.* 8 113–124.

